# The Prevalence of Anxiety and Depression Symptoms in Obstructive Sleep Apnea

**DOI:** 10.7759/cureus.11203

**Published:** 2020-10-27

**Authors:** Wahida Akberzie, Sean Hesselbacher, Ishan Aiyer, Salim Surani, Zoya S Surani

**Affiliations:** 1 Family Medicine, Eastern Virginia Medical School, Norfolk, USA; 2 Medicine, Eastern Virginia Medical School, Norfolk, USA; 3 Medicine, Hampton Veterans Affairs Medical Center, Hampton, USA; 4 Sleep Medicine, Torr Sleep Center, Corpus Christi, USA; 5 Internal Medicine, Texas A&M University, Corpus Christi, USA; 6 Internal Medicine, Torr Sleep Center, Corpus Christi, USA

**Keywords:** obstructive sleep apnea, anxiety, depression

## Abstract

Objectives

Mood disorders are common in obstructive sleep apnea (OSA), though the interactions are not well-understood. The objective of this study was to evaluate the relationship between anxiety and depression with OSA.

Methods

Patients who presented to the sleep center underwent polysomnography (PSG). Records were included if the sleep study showed OSA (Apnea-Hypopnea Index (AHI) ≥5 events/hour). All patients completed an Epworth Sleepiness Scale (ESS) and Hospital Anxiety and Depression Scale (HADS). A score of 8 or higher on the respective portion of the HADS was abnormal.

Results

A total of 45 records were included, with 28 scoring positive for anxiety and 29 positive for depression. Patients with anxiety had lower AHI (median (interquartile ratio)) than those without (21.4 (9.6-41.3) vs. 50.5 (25.1-94.3); p=0.0076). The peripheral oxygen saturation (SpO_2_) nadir (80 (74-84)% vs. 65 (57-76)%; p=0.0007) and time with SpO_2_ <90% (11 (6-12) minutes vs. 36 (13-68) minutes; p=0.0002) were less abnormal in patients with anxiety. The anxiety score on the HADS weakly correlated with AHI (r = -0.29). Patients with depression were not significantly different than those without depression in AHI, SpO_2_ nadir, and time with SpO_2_ <90%.

Conclusions

Symptoms of anxiety and depression are both prevalent in patients with OSA. There is an inverse relationship between OSA severity and the presence of anxiety, suggesting that comorbid anxiety may prompt sleep evaluation in less severe disease. Depression symptoms did not demonstrate a similar relationship with OSA severity.

## Introduction

Obstructive sleep apnea is a sleep-related breathing disorder in which a person has a decrease or complete obstruction of airflow during his or her sleep. OSA occurs when muscles relax during sleep and soft tissue of the throat collapse and block the upper airway [1]. This can lead to partial reductions in airflow with resultant desaturation of ≥4% (hypopneas) and complete pauses (apneas) in breathing during sleep [2]. Common symptoms of OSA include fatigue and daytime sleepiness. The prevalence of OSA with accompanying daytime sleepiness is approximately 3 to 7% for adult men and 2 to 5% for adult women in the general population based on available population-based studies [3].

Obstructive sleep apnea is known to have significant neuropsychiatric manifestations [4,5]. These may be related to both the neurobehavioral effects of intermittent hypoxia [6] as well as the effects of sleep fragmentation [7]. The associations between anxiety/depression and obstructive sleep apnea are not well understood. Some studies have found that patients with OSA had more depressive symptoms than those without OSA, but that depressive symptoms were associated with poor sleep quality rather than OSA severity [8]. However, OSA goes undiagnosed in many individuals. Therefore, identifying individuals with psychiatric illness and OSA may help prevent long-term detrimental health effects.

The main objective of this study was to evaluate the relationship of anxiety and/or depression with OSA. This includes assessing the correlation of depression and anxiety with OSA severity. We hypothesized that individuals with more severe obstructive sleep apnea would have more severe symptoms of anxiety and depression.

## Materials and methods

Study design

Patients undergoing polysomnography (PSG), from May through September 2006, at the Baylor College of Medicine Sleep Center in Houston, Texas were provided with the Hospital Anxiety and Depression Scale (HADS) to complete, in addition to standard pre-sleep study paperwork. Inclusion criteria were the presence of obstructive sleep apnea (apnea-hypopnea index, AHI ≥5 events per hour) on a diagnostic or split-night sleep study, completion of the HADS questionnaire, and age >18 years. All records meeting inclusion criteria were selected for review; records were excluded if the patient failed to complete either the HADS or PSG. All procedures performed in studies involving human participants were in accordance with the ethical standards of the institutional and/or national research committee and with the 1964 Helsinki declaration and its later amendments or comparable ethical standards.

Questionnaires

At the time of PSG testing, each patient was provided questionnaires to complete, including demographic questions (including self-reported age, gender, and ethnicity), the Epworth Sleepiness Scale (ESS) [9] and the HADS [10]. The HADS questionnaire is a validated 14-item screen for anxiety and/or depression; each item is answered with a number 0-3 based on the frequency of occurrence of a feeling over the past week. The questionnaire is comprised of two parts: a seven-item screen for anxiety (HADS-A) and a seven-item screen for depression (HADS-D); the total score for each part results in a normal (0-7), borderline (8-10), or abnormal (11-21) screen for anxiety/depression. As detailed earlier, both sensitivity and specificity of HADS-A and HADS-D approximate 80% [11]. In a specialized population, it was found that a HADS-D score ≥8 has a positive predictive value (PPV) of 67.57% and a negative predictive value (NPV) of 92.19%; a HADS-A score ≥11 has a PPV of 71.05% and a NPV of 96.83% [12].

Polysomnography

Sleep studies were performed using attended comprehensive PSG, including recording electroencephalogram, electrooculogram, submentalis electromyogram, airflow, respiratory effort, oxygen saturation, anterior tibialis electromyogram, and heart rhythm. Recordings were scored by a technologist manually according to the American Academy of Sleep Medicine Scoring Guidelines [13] and interpreted by a board-certified sleep physician.

Statistics

Comparisons between two non-normally distributed groups were done with the Mann-Whitney U test. The normality of a group distribution was determined using D’Agostino-Pearson omnibus normality test. Two groups of dichotomous variables were compared with the Fisher’s exact test. Correlation analysis was performed by calculating the Spearman correlation coefficient. A p-value of <0.05 was considered statistically significant.

## Results

**Figure 1:**
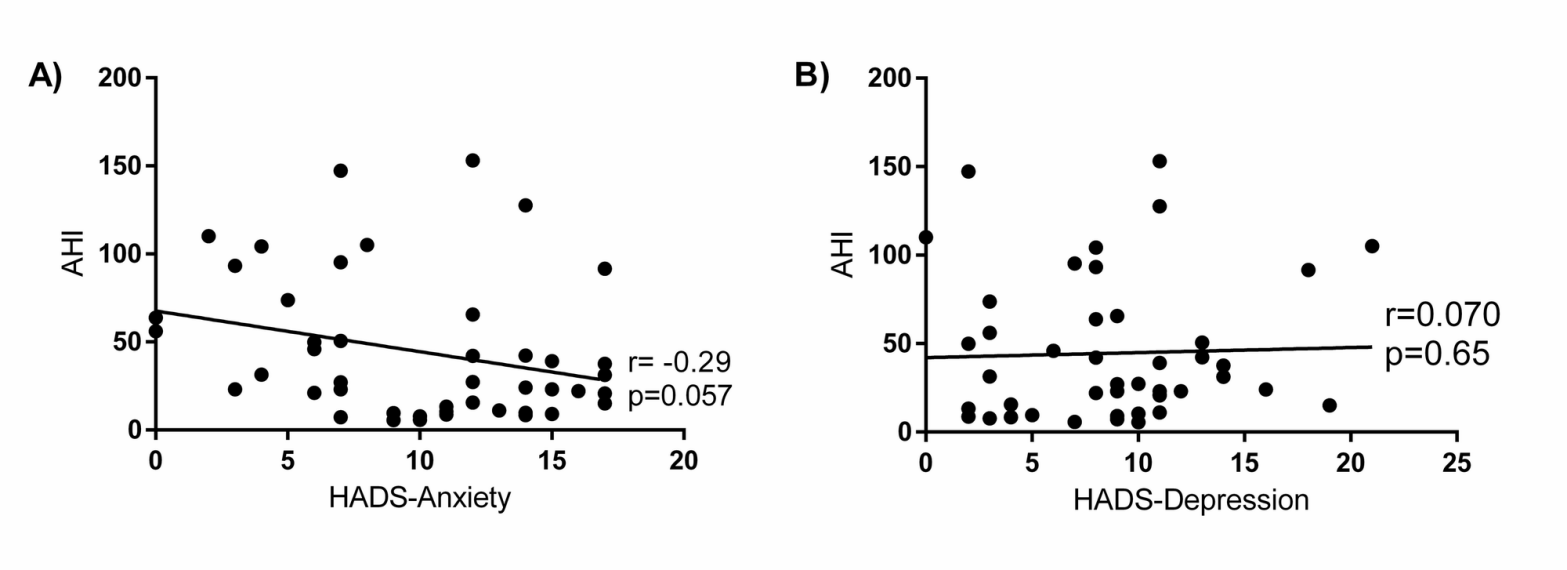
Apnea-Hypopnea Index (AHI) is weakly negatively correlated with the Hospital Anxiety and Depression Scale (HADS)-Anxiety score (A) but not HADS-Depression score (B).

A total of 45 records were included in this study, of which 29 were females and 16 males. The median age was 47 (Table 1). A total of 28 individuals scored positive for anxiety and 29 were positive for depression. The demographics and characteristics comparing those with and without anxiety symptoms are shown in Table 2. Patients with anxiety symptoms had lower AHI (median (interquartile ratio, IQR)) than those without anxiety (21.4 (9.6-41.3) vs. 50.5 (25.1-94.3); p=0.0076), respectively. The peripheral oxygen saturation (SpO_2_) nadir was higher in the anxiety group compared to the non-anxiety group (80 (74-84)% vs. 65 (57-76)%; p=0.0007). Also, time with SpO_2_ <90% was less in the anxiety group compared to the group without anxiety (11 (6-12) minutes vs. 36 (13-68) minutes; p=0.0002), respectively. Figure 1A displays the correlation of HADS-A score with AHI. The correlation coefficient (r) was found to be -0.29 with a p-value of 0.057.

**Table 1:** Demographic and clinical characteristics of the participants Abbreviations: IQR = interquartile ratio; BMI = body mass index; ESS = Epworth Sleepiness Scale; AHI = apnea-hypopnea index; SpO2 = peripheral oxygen saturation *Race was not reported by one participant

Number	45
Age (years), median (IQR)	47 (41 – 52)
Male, n (%)	16 (36%)
Race, n (%)*	
Hispanic	15 (34%)
Caucasian	12 (27%)
African American	17 (39%)
BMI (kg/m^2^), median (IQR)	42.5 (37.9 – 48.0)
ESS score, median (IQR)	11 (7 – 16)
AHI (events/hour), median (IQR)	27.3 (12.2 – 64.7)
SpO_2_ nadir (%), median (IQR)	76 (65.5 – 83)%
Minutes with SpO_2_ <90%, median (IQR)	11.0 (10.0 – 46.1)

**Table 2:** Association between obstructive sleep apnea (OSA) severity and anxiety Abbreviations: HADS-A = Hospital Anxiety and Depression Scale anxiety score; IQR = interquartile ratio; BMI = body mass index; ESS = Epworth Sleepiness Scale; AHI = apnea-hypopnea index; SpO2 = peripheral oxygen saturation *p<0.05 **Race was not reported by one participant

	No Anxiety (HADS-A <8)	Anxiety (HADS-A ≥8)	P-value
Number	17	28	
Age (years), median (IQR)	47 (39 – 52)	43 (31 – 49)	0.52
Male, n (%)	7 (41%)	9 (32%)	0.75
Race, n (%)**			
Hispanic	6 (35%)	9 (33%)	0.53
Caucasian	6 (35%)	6 (22%)	
African American	5 (29%)	12 (43%)	
BMI (kg/m^2^), median (IQR)	42.8 (39.6 – 59.1)	41.2 (35.7 – 46.4)	0.11
ESS score, median (IQR)	14 (7 – 18)	10 (7 – 15)	0.49
AHI (events/hour), median (IQR)	50.5 (25.1 – 94.3)	21.4 (9.6 – 41.3)	0.0076*
SpO_2_ nadir (%), median (IQR)	65 (57 – 76)	80 (74 – 84)	0.0007*
Minutes with SpO_2_ <90%, median (IQR)	36 (13 – 68)	11 (6 – 12)	0.0002*

Table 3 compares the characteristics of the participants with and without depression symptoms. Individuals with and without depression symptoms had similar median (IQR) AHI of 27.3 (20.9-64.7) vs. 23.5 (8.9-69.4); p=0.51. The SpO_2_ nadir was higher in the depression group vs. the group without depression (77 (69-84) vs. 75 (56-82)), respectively. Time with SpO_2_ <90% was lower in the depression group compared to the non-depression group with 11 (9-45) minutes and 13 (10-46) minutes, respectively and a p-value=0.95. The correlation of HADS-D score and AHI is displayed in Figure 1B. The correlation coefficient was 0.07 and a p-value of 0.65. 

**Table 3:** Association between obstructive sleep apnea (OSA) severity and depression Abbreviations: HADS-D = Hospital Anxiety and Depression Scale depression score; IQR = interquartile ratio; BMI = body mass index; ESS = Epworth Sleepiness Scale; AHI = apnea-hypopnea index; SpO2 = peripheral oxygen saturation *Race was not reported by one participant

	No Depression (HADS-D <8)	Depression (HADS-D ≥8)	P-value
Number	16	29	
Age (years), median (IQR)	47 (38 – 53)	47 (43 – 52)	0.75
Male, n (%)	7 (44%)	9 (31%)	0.52
Race, n (%)*			
Hispanic	9 (56%)	6 (21%)	0.52
Caucasian	3 (19%)	9 (32%)	
African American	4 (25%)	13 (46%)	
BMI (kg/m^2^), median (IQR)	42.7 (37.8 – 45.8)	42.5 (37.7 – 52.8)	0.75
ESS score, median (IQR)	11 (8 – 14)	12 (7 – 19)	0.56
AHI (events/hour), median (IQR)	23.5 (8.9 – 69.4)	27.3 (20.9 – 64.7)	0.51
SpO_2_ nadir (%), median (IQR)	75 (56 – 82)	77 (69 – 84)	0.23
Minutes with SpO_2_ <90%, median (IQR)	13 (10 – 46)	11 (9 – 45)	0.95

## Discussion

In this study, there was a high prevalence of co-morbid anxiety and depression in OSA (62.2% and 64.4%, respectively). Participants with and without anxiety were not statistically different in terms of age, gender, race, BMI, or ESS score. The group with anxiety symptoms had a statistically significant lower AHI compared to the group without anxiety symptoms. The SpO_2_ nadir was statistically higher in the group with anxiety symptoms. Lastly, the time with SpO_2_ <90% was significantly different between the groups with and without anxiety symptoms, with less time spent with SpO_2_ <90% in the anxiety group. These findings suggest that patients with anxiety using HADS-A scoring had less severe OSA findings. The trend of AHI with HADS-A score shows as severity of anxiety increases, AHI severity decreased, resulting in a negative correlation coefficient.

A large study by Bjorvatn et al. included a sample of 3770 patients in Norway evaluating severity of OSA and association with anxiety and depression. This study also used HADS-A (HAD-A ≥8) to evaluate for anxiety. It was found that there were fewer individuals with anxiety as severity of OSA increased [14]. Lee et al. looked at apnea severities and severity of anxiety with State-Trait Anxiety Inventory in Korea, and found the prevalence of anxiety was highest in patients with mild OSA and lowest in patients with severe OSA [15]. The reasoning of the inverse relationship between anxiety and OSA severity is not clear. Animal models have suggested that intermittent hypoxia may reduce behaviors of anxiety [16, 17]; pathological findings in these studies included lower amyloid beta levels in the cortex and hippocampus, and protection against increased striatal catecholamines seen during sleep deprivation. Clinically, patients with anxiety disorder may be more sensitive to the sleep disturbances and/or intermittent hypoxia caused by even mild OSA, thus seeking out evaluation.

Patients with depression were not significantly different than those without depression in age, gender, race, BMI or ESS score, AHI, SpO_2_ nadir, and time with SpO_2_ <90%. Also, SpO_2_ nadir and time with SpO_2_ <90% were not statistically different in the depression and no-depression group. Their correlation between HADS-D score and AHI was positive but weak. 

Bjorvatn et al. found depression (using HADS-D) was not related significantly with OSA severity [14]. Lee et al. used Beck Depression Inventory and State and Trait Anxiety Inventory to assess depression severity in patients and found that depressive symptoms were more prevalent in patients with mild OSA than those with severe OSA [15]. Garbarino et al. conducted a systemic review of literature that included 73 articles, looking at the association of anxiety and depression with severity of OSA. It was observed that most literature does not support the existence of an association between OSA severity and the presence of depressive symptoms or anxiety, although there was a limited number of articles that included the data of interest [18]. The lack of a clear relationship between depression and OSA severity may be due to several factors.

Our current study is limited by the small sample size from a single institution, affecting the power and generalizability of the findings. The HADS was developed for identifying cases of anxiety and depression among patients in non-psychiatric hospital clinics, but not as a formal diagnostic tool; it has been found to be valid when used in community settings and primary medical practice [19]. In the future, it may be helpful to include patients that do not have OSA. Measurable sleep factors other than sleep-disordered breathing, such as sleep efficiency or sleep fragmentation, likely impact mood and should be considered in future studies. Evaluating patients for a prior history of anxiety or depression and obtaining medication history may help investigate for confounding factors. In recent years, other studies looking at the prevalence of psychiatric illness in OSA patients. Knechtle et al. looked at individuals between 2016 and 2018 and found the prevalence of moderate to severe OSA to be 72.48% in patients with a serious psychiatric disease (major depressive disorder, bipolar disorder, schizophrenia, or a psychotic disorder) using Diagnostic and Statistical Manual of Mental Disorders diagnostic criteria [20]. While in Garbarino’s meta-analysis a pooled prevalence of depressive and anxious symptoms in OSA patients was found to be 35% and 32%, respectively [18]. Therefore, it may be difficult to assess prevalence of anxiety and depression in the OSA population as several symptoms can overlap. In some reports, it was thought that there is an overestimation of depression and anxiety in OSA as it can be difficult distinguishing the sleep disorder from psychiatric illness [18]. Until the relationship is better understood, it may be difficult to quantitate exactly how many individuals suffering from OSA who also coincidentally suffer from depression or anxiety.

## Conclusions

This study highlights the need for continued evaluation of the associations between depression and anxiety with obstructive sleep apnea. The inverse relationship between anxiety severity and OSA severity, in particular, is curious and warrants further investigation. Understanding these relationships may provide essential information to better treat patients and help identify patients that may have underlying psychiatric disease.
